# Drinking Patterns and Alcohol Use Disorders in São Paulo, Brazil: The Role of Neighborhood Social Deprivation and Socioeconomic Status

**DOI:** 10.1371/journal.pone.0108355

**Published:** 2014-10-01

**Authors:** Camila Magalhães Silveira, Erica Rosanna Siu, James C. Anthony, Luis Paulo Saito, Arthur Guerra de Andrade, Andressa Kutschenko, Maria Carmen Viana, Yuan-Pang Wang, Silvia S. Martins, Laura Helena Andrade

**Affiliations:** 1 Section of Psychiatric Epidemiology - LIM 23, Institute of Psychiatry, School of Medicine, University of São Paulo, São Paulo, São Paulo, Brazil; 2 Program of the Interdisciplinary Group of Studies on Alcohol and Drugs (GREA), Department and Institute of Psychiatry, School of Medicine, University of São Paulo, São Paulo, São Paulo, Brazil; 3 Department of Epidemiology and Statistics, Michigan State University, East Lansing, Michigan, United States of America; 4 Department of Epidemiology, Mailman School Of Public Health, Columbia University, New York, New York, United States of America; Centre for Addiction and Mental Health, Canada

## Abstract

**Background:**

Research conducted in high-income countries has investigated influences of socioeconomic inequalities on drinking outcomes such as alcohol use disorders (AUD), however, associations between area-level neighborhood social deprivation (NSD) and individual socioeconomic status with these outcomes have not been explored in Brazil. Thus, we investigated the role of these factors on drink-related outcomes in a Brazilian population, attending to male-female variations.

**Methods:**

A multi-stage area probability sample of adult household residents in the São Paulo Metropolitan Area was assessed using the WHO Composite International Diagnostic Interview (WMH-CIDI) (n = 5,037). Estimation focused on prevalence and correlates of past-year alcohol disturbances [heavy drinking of lower frequency (HDLF), heavy drinking of higher frequency (HDHF), abuse, dependence, and DMS-5 AUD] among regular users (RU); odds ratio (OR) were obtained.

**Results:**

Higher NSD, measured as an area-level variable with individual level variables held constant, showed an excess odds for most alcohol disturbances analyzed. Prevalence estimates for HDLF and HDHF among RU were 9% and 20%, respectively, with excess odds in higher NSD areas; schooling (inverse association) and low income were associated with male HDLF. The only individual-level association with female HDLF involved employment status. Prevalence estimates for abuse, dependence, and DSM-5 AUD among RU were 8%, 4%, and 8%, respectively, with excess odds of: dependence in higher NSD areas for males; abuse and AUD for females. Among RU, AUD was associated with unemployment, and low education with dependence and AUD.

**Conclusions:**

Regular alcohol users with alcohol-related disturbances are more likely to be found where area-level neighborhood characteristics reflect social disadvantage. Although we cannot draw inferences about causal influence, the associations are strong enough to warrant future longitudinal alcohol studies to explore causal mechanisms related to the heterogeneous patterns of association and male-female variations observed herein. Hopefully, these findings may help guide future directions for public health.

## Introduction

Alcohol consumption is a leading risk factor for global disability-adjusted life years (DALYs). In 2010, alcohol use was the 5th ranked DALYs determinant at the global level and was in the 1st rank for parts of Latin America, Eastern Europe, and southern sub-Saharan Africa [1]. Moreover, alcohol ranks as a major determinant of non-communicable diseases, especially in middle-income countries [2], [3]. In Brazil, the largest middle-income country in Latin America and the site of the São Paulo Megacity Mental Health Survey, alcohol consumption represents a significant burden. In 2004, countries were ranked by size of alcohol-attributable DALYs affecting men: Russia was top-ranked at #1 on the list; Brazil was #2. Among women, the alcohol-attributable DALYs placed Brazil at #3 behind Russia and the USA [4].

The worrisome impact of alcohol use on health outcomes in Brazil might derive from separately identifiable drinking patterns and alcohol-related disturbances such as alcohol use disorders (AUD). These, in turn, are subject to both individual-level and society-level influences, including macro area-level contextual influences [5]–[7]. For instance, the top 10% of drinkers by volume in Brazil drink about half of all alcohol consumed in the country [8]. An estimated 12% of the adult general population in Brazil meet criteria for lifetime history of AUD [9]–[11]. Brazil’s rather permissive drinking culture is reflected in data showing that most alcohol users initiate alcohol consumption when they are 17 years old [9] or even earlier [10], [12], [13] and also drink in risky patterns [10], [12], [14]–[16]. Furthermore, Brazil ranks well above many other countries on the 5-point harmful drinking score created for the Comparative Risk Assessment module of the Global Burden of Diseases project in an effort to estimate how changes in population health might depend upon harmful drinking. Brazil’s summary score value of three out of five is based on its relatively high position on indicators such as frequency of drinking, frequency of heavy drinking occasions, usual quantity of alcohol consumed per occasion, drinking in public settings and during festive events, proportion of drinking events when drinkers get drunk, proportion of drinkers who drink daily or nearly daily, and only drinking with meals [17]–[19].

As for male-female variations, as is true in many countries, Brazilian women are more likely to abstain from alcohol. Other research has shown that males in the total population (including abstainers) are more than twice as likely as women to be heavy drinkers or to meet criteria for AUD [15], [16], [20]–[23]. Distinctive and innovative features of this study include diversity of neighborhoods within the megacity and the study team’s attempt to shed light on male-female variations in the associations between alcohol outcomes with individual-level facets of socioeconomic status (SES), such as income, employment and educational attainment [22], [24]–[27], within the context of a conceptual model that holds constant macro area-level neighborhood social deprivation (NSD) using source of data that are external and not reliant on the responses of the survey participants [28]. For the first time in Brazil, and for males and for females separately, we have estimated associations linking alcohol outcomes with independently derived area-level NSD scores, within a conceptual model that holds constant potentially influential individual-level variables.

We are not the first to study SES in relation to drinking outcomes, and our study has the character of an initial exploratory step in Brazil, with a primary focus on whether there might be statistically robust associations linking higher NSD with greater occurrence of heavy drinking and other alcohol-related disturbances (such as AUD) among drinkers who have consumed at least 12 drinks in the past year. In prior studies, from other countries, there is evidence that social position is associated with alcohol use and related problems [29]–[33], but alcohol consumption does not seem to follow the conventional pattern of lower socioeconomic groups having worse health than those at higher SES [31], [34]. Previous research in North America found that household income, education, and employment status were positively associated with current and frequent drinking, but were negatively associated with heavy drinking and AUD (e.g. [34], [35]). Hemmingsson’s research group [36] found comparatively more diversity among suspected causal influences for alcohol dependence among lower SES and unemployed individuals. Conversely, research from low-middle income countries and from countries in transition such as Russia found higher SES to be positively associated with AUD and problem drinking [37].

Attempts to integrate lines of sex differences research with lines of SES research add some complexity. In higher income countries, well-educated professional women sometimes have been found with equal or greater occurrence of heavy drinking and alcohol problems as compared to men [38], [39]. Adding more complexity, research conducted in high-income countries recently discloses evidence of potential neighborhood–level deprivation effects on drinking patterns and AUD, which might vary across the sexes [40], [41]. Nonetheless, in general, living in less deprived neighborhoods has been associated with being an alcohol drinker [42], [43] and regularly using alcohol [44], while living in more deprived areas has been associated with abstinence from alcohol [45], heavy drinking [45], [46], and alcohol-related problems [45], [47].

We designed this project with a focus on investigating male-female variations in associations linking various alcohol outcomes with neighborhood social deprivation measured as a macro characteristic of areas of residence, with an emphasis on active alcohol-related disturbances (heavy drinking through AUD) among adults with at least 12 drinks in the past year. We also offer estimates for prevalence of alcohol outcomes in Brazil, in an exploration of those more basic epidemiological topics. To clarify which NSD-alcohol associations might be strong enough to warrant future prospective or longitudinal research to build upon this initial foundation of cross-sectional data, our modeling of these outcomes allows for statistical control of individual-level covariates (e.g., SES) when estimating the NSD associations. We understand that the cross-sectional character of the data from Brazil means that we can draw no firm causal inferences, but what is most interesting to us is whether there is a consistently observed and sufficiently large association linking area-level NSD with alcohol-related disturbances among recently active drinkers, even with individual-level covariates held constant. If there is no robust NSD association with these alcohol outcomes, then research planning should be directed toward other facets of neighborhood context beyond the boundaries set for the area-level NSD construct as studied in this project.

## Materials and Methods

### Survey characteristics and study population

The “São Paulo Megacity Mental Health Survey” (SPMHS) is part of the World Mental Health Survey Initiative (WMHS), which was launched by WHO in 2000 and has been carried out in 28 countries with similar methodology. The present study assessed a probabilistic sample of household residents aged 18 years or older in the São Paulo Metropolitan Area (SPMA), which is composed by 38 municipalities and the city of São Paulo, Brazil. A detailed overview of the survey, including aims, design, sampling procedures and field implementation, has been reported elsewhere [48].

Eligible respondents were selected from a stratified multistage-clustered area probability sample of households. In all strata, the primary sampling units (PSUs) were 2,000 census count areas, according to updated geographical definitions of the Instituto Brasileiro de Geografia e Estatística (IBGE – Brazilian Institute of Geography and Statistics) [49]. The 38 municipalities composed 60% of the total sample, with municipalities being self-representative and contributing to the total sample size proportional to their population density. In complement, the city of São Paulo, formed by five regions with 96 PSUs, contributed to 40% of the total sample. Within each sampled household, one respondent per dwelling was sampled by a Kish selection table [50].

The total observed sample consisted of 5,037 individuals, with a summary participation level of 81%. Before fieldwork, lay-interviewers received a 7-day standardized training by the Principal Investigators (LHA and MCV). For all respondents, face-to-face interviews were conducted between May/2005 and April/2007, after signing a written informed consent.

### Ethics Statements

The SPMHS procedures for recruitment, obtaining informed consent, and protecting human subjects during field procedures were approved by the Research and Ethics Committee of the University of São Paulo Medical School. Respondents were interviewed only after informed written consent was obtained, and total confidentiality was assured. Eligible respondents were those who were 18 or older, Portuguese-speaking, and without any disability or handicap that would otherwise impair their ability to participate in the interview.

### Assessment procedures

The WMH version of the Composite International Diagnostic Interview 3.0 (WMH-CIDI 3.0) was translated and adapted to the Brazilian-Portuguese language [48]. The WMH-CIDI 3.0 is a fully structured diagnostic interview that generates psychiatric diagnoses according to both ICD-10 (International Statistical Classification of Diseases and Related Health Problems, 10th revision) and DSM-IV criteria [51], [52].

The WMH-CIDI has clinical and non-clinical modules distributed across Part 1 and Part 2 sections, with ‘core’ psychiatric disturbances assessed in Part 1. Part 1 is administered to all WMH respondents. Based on Part 1 responses, those who meet criteria for lifetime history of core disturbances, plus a 25% random sample of all others, are asked to complete Part 2 modules, which include non-clinical modules and non-core diagnostic assessments.

The alcohol module of WMH-CIDI 3.0 is in Part 1 so that all participants answered questions regarding alcohol use, drinking patterns, and related disturbances. Those who consumed at least one drink in the previous year are termed ‘past-year users’. Across a broad range from the most frequent alcohol consumers to those who consumed at least 12 drinks in the previous 12 months, we have a heterogeneous subgroup of past year drinkers, distinguished with the somewhat arbitrary term ‘regular user’ (RU) [9], [53]–[55]. Within this RU subgroup, three mutually exclusive subgroups were formed to distinguish between (1) heavy drinkers of lower frequency (HDLF, sometimes termed ‘heavy episodic drinkers’) who have consumed five or more drinks in a row for men and four or more drinks in a row for women, but no more often than two times per month; (2) heavy drinkers of higher frequency for whom heavy drinking occurs at least three times per month (HDHF). Alcohol use disorders qualify as separate alcohol-related disturbances among regular users, and were identified via the WMH-CIDI and its diagnostic algorithm’s application of both DSM-IV (abuse and dependence), independently assessed via the ‘ungated’ approach described in prior papers [9], [56]) and DSM-5 criteria (for AUD: alcohol use disorders) diagnoses.

Many of this study’s prevalence analyses are ‘conditional’ in that they restrict the denominator of each proportion to ‘past-year drinkers’, while other analyses are ‘conditional’ because the denominator is restricted to individuals who had consumed at least 12 drinks in the past year (RU); all others are assumed to be effectively not at risk for being an active heavy drinker or for qualifying as a case of a DSM-IV or DSM-5 alcohol disorder in the past year.

An alternative approach is used when the goal is to produce total population estimates for alcohol outcomes that are directly comparable to total population estimates for some other condition (e.g., cannabis outcomes), in which case the denominator for the proportion is the total population, and the resulting estimates can be used to derive an estimated count of cases in the population who might need alcohol treatment services or cannabis treatment services. Here, we have restricted some denominators to ‘past year drinkers’ and to ‘regular drinkers’ so as to understand the relative occurrence and patterns of association of alcohol outcomes among the recently active users, as explained in footnotes to the tables. For example, for the ‘conditional prevalence’ of RU among past-year drinkers, the denominator excludes lifelong abstainers as well as those who drank in past years but have not had a drink in the past year. Further in ‘conditional prevalence’ analyses restricted to regular users, the interpretation of the estimates involves thinking about how many of the current regular users now qualify as cases of heavy drinking, or DSM-IV abuse, dependence or DSM-5 AUD.

### Covariates

The key covariate focus is on the macro area-level NSD values related to alcohol outcomes, with individual-level SES held constant via terms for education, employment status, and income. Regression models also held constant age (18–34, 35–54, 55 years or more) and marital status (never married; previously married; married or cohabiting); for total sample (men and women combined), sex was held constant as well. For women with DSM-IV abuse, dependence or DSM-5 AUD age strata were divided into two subgroups of 18–34, 34–54 because no woman over the age of 55 years filled criteria for these diagnoses.

Education was coded by years of schooling: 0–4 (low); 5–8 (low-average); and 9+ (high-average and high). Employment status was coded as (1) workers paid outside the household and students, (2) unemployed, and (3) retired or working as a homemaker in one’s own household.

For income, the standard international labor economics method was used [57], with per capita income calculated by dividing total household income by the number of household members. Income levels were defined according to the per capita income in comparison with the Brazilian median per capita income (7,050 dollars/year): low (less than half the Brazilian median), low-average (more than half of the Brazilian median up to the median), high-average (above the Brazilian median up to three times the median), and high (above three times the Brazilian median).

The area-level NSD variable was developed by the Center of Metropolitan Studies (http://www.centrodametropole.org.br) and assigned to each census unit, to reflect social conditions in the SPMA geographical space using data from the 2000 Census. This index, derived from external census sources, combines socioeconomic deprivation indicators (income, level of education, family size, and percentage of families headed by a woman with low educational level) and the population’s age structure. The NSD index ranges from 1 (no social deprivation) to 8 (high social deprivation). These eight levels were summarized in 3 indicators: no-low (combined index of 1, 2, and 3 NSD level), medium-low/medium (6 and 4), and high/very high NSD (5, 7, and 8).

### Data analysis

Since data were obtained from a complex stratified sample design, sample weights and design variables that account for sample clustering were applied. Prior to analysis, all respondents received a pre-stratification weight to adjust for within household and PSU probabilities of selection, and a post-stratification weight to adjust for the known age and sex structure of the SPMA population and non-response [48].

The analysis weight for the World Mental Health Surveys initiative, for this study’s prevalence estimates, and for this study’s regression analyses is based on (a) the inverse of the probability of selection into the sample, and (b) a post-stratification adjustment factor such that, after weighting, the survey-based estimates for basic demographic distributions (e.g., age, sex) serve well as population projections for the São Paulo Megacity Mental Health Survey population (as best they are known from the most recent corresponding census distributions for the São Paulo Metropolitan Area that was surveyed). The application of these analysis weights in the survey analyses, via SAS procedures, are as described in Chapter 4 of a recently published textbook authored by WMHS collaborators [58].

Data analyses were conducted using the SAS software version 9.1 (SAS, 2004) and SUDAAN (Research Triangle Institute, 2004). Cross-tabulations were used to estimate overall prevalence of recently active (past-year) drinking, as well as ‘conditional prevalences’ for the other alcohol outcomes. Male-female variations in associations were estimated via stratified analyses.

Quantity and frequency of alcohol consumption in the last 12 months were determined for non-heavy drinkers, heavy drinkers of lower frequency and heavy drinkers of higher frequency by sex.

Bivariate analyses and multiple logistic regression were used to estimate area-level NSD associations with alcohol outcomes, and then to explore associations with covariates, with secondary focus on the SES indicators, holding area-level NSD constant and vice versa. For HDLF and HDHF, polychotomous logistic regression was performed with non-heavy drinking as the reference category.

Odds ratios (OR) were estimated for NSD and the other covariates, first in unadjusted form with Wald tests [59]. Thereafter, OR estimation was with covariate adjustment to obtain a final multivariate model. For total sample analysis, models were adjusted for both age and sex; while for male-female stratified analyses were adjusted for age. Significance testing, standard errors (SE) and 95% confidence intervals (95% CI) were estimated using Taylor series linearization methods for complex sample data [60], as implemented in SUDAAN (Research Triangle Institute, 2004). Multivariate significance tests were conducted with Wald χ^2^ tests using Taylor series design-based coefficient variance-covariance matrices. All significance tests were based on two-sided tests at a 0.05 significance level.

## Results

### Study Sample

Socio-demographic characteristics of the study sample have been described elsewhere [48]. Briefly, there were more women (57%) than men in the sample, and it was a relatively young sample: 60% of the subjects aged less or equal to 45 years old; 71% of the men and 60% of the women were married. Approximately half of the subjects had low/low-average education and around 60% were employed (76% of men, 50% of women). Table S1 provides the full distribution of the NSD variable based on unweighted sample statistics, a distribution based on application of the analysis weights, (i) for the sample as a whole, and (ii) for males and for females, separately.

### Prevalences of alcohol use and outcomes: unconditional and conditional

Table 1, bottom half, shows unconditional prevalence of recently active (past-year) drinking, as well as the estimated conditional prevalence for all recently active alcohol outcomes. Overall, an estimated 46% of the São Paulo adult population had consumed alcohol in the past year. Among past year drinkers, overall, 71% qualified as ‘regular users’, having consumed at least 12 drinks in the past year.

**Table 1:** Estimated subgroup-specific prevalence proportions for recently active alcohol use, drinking patterns and related disturbances.

Characteristics	N	Past-yearuse (n = 2180)			Regularuse^1^ (n = 1513)			Among Regular Drinkers									Abuse^2^(n = 125)			Dependence^2^(n = 59)			DSM-5 AUD^2^(n = 132)		
								Non-heavydrinking^2^(n = 1105)			Heavydrinkingof lowerfrequency^2^(n = 111)			Heavy drinkingof higherfrequency^2^(n = 297)											
		n	% (SE)	*P* (χ2)	n	% (SE)	*P* (χ2)	n	% (SE)	*P* (χ2)	n	% (SE)	*P* (χ2)	n	% (SE)	*P* (χ2)	n	% (SE)	*P* (χ2)	n	% (SE)	*P* (χ2)	n	% (SE)	*P* (χ2)
**Age. years**				<0.0001			0.5676			<0.0001			<0.0001			<0.0001			<0.0001			0.7892			0.0939
18–34	1841	879	49.8 (1.2)		579	69.7 (2.1)		378	61.9 (2.4)		77	14.2 (1.3)		142	24.0 (2.0)		59	9.5 (1.5)		22	4.0 (1.1)		54	8.8 (1.6)	
35–54	2160	966	46.6 (1.9)		676	72.4 (2.1)		511	75.5 (2.2)		29	4.3 (1.2)		136	20.3 (2.4)		60	8.2 (1.4)		33	4.0 (0.8)		70	8.9 (1.3)	
55+	1036	335	32.6 (2.2)		240	72.9 (3.8)		216	92.0 (2.4)		5	1.3 (0.6)		19	6.7 (2.0)		6	1.2 (0.6)		4	2.4 (1.8)		8	3.2 (1.9)	
**Sex**				<0.0001			<0.0001			0.0884			0.9111			0.0185			0.0927			0.0948			0.0708
Men	2187	1330	61.8 (1.2)		1040	78.6 (1.5)		744	69.1 (1.9)		77	8.7 (1.0)		219	22.2 (1.5)		102	6.3 (1.2)		45	4.4 (.7)		108	9.1 (1.0)	
Women	2850	850	31.3 (1.1)		473	58.1 (2.2)		361	75.6 (2.5)		34	8.4 (2.0)		78	15.9 (2.1)		23	1.7 (1.4)		14	2.5 (.6)		24	5.9 (1.4)	
**Marital status**				0.0343			0.7217			0.0271			0.0172			0.3287			0.3282			0.8687			0.2073
Married/cohabiting	3250	1397	45.0 (1.1)		981	72.0 (1.4)		736	74.8 (2.0)		61	6.2 (1.0)		184	19.0 (1.6)		74	7.1 (1.2)		37	3.5 (.7)		78	7.1 (1.1)	
Previously married	894	353	42.0 (1.8)		238	70.2 (4.6)		179	73.4 (2.7)		13	7.3 (2.9)		46	19.4 (2.5)		18	7.0 (1.7)		11	4.2 (1.4)		20	7.1 (1.6)	
Never married	893	430	49.3 (1.4)		294	69.9 (2.5)		190	61.7 (3.4)		37	14.6 (2.3)		67	23.7 (2.8)		33	10.2 (2.0)		11	4.2 (1.6)		34	10.8 (1.9)	
**Education**				<0.0001			0.9946			0.5539			<.0001			0.7751			0.6995			0.1088			0.0634
Low	1344	453	34.9 (1.8)		314	71.0 (2.8)		239	74.4 (3.8)		11	3.4 (1.2)		64	22.2 (4.4)		32	8.6 (1.6)		25	7.8 (1.8)		42	11.4 (2.4)	
Low-average	1262	546	45.8 (1.4)		387	71.0 (1.8)		289	72.4 (3.5)		23	6.0 (1.5)		75	21.6 (3.4)		34	8.9 (2.2)		14	3.4 (1.4)		39	9.8 (2.2)	
High-average+High	2431	1181	49.9 (0.9)		812	71.3 (2.0)		577	69.5 (1.4)		77	11.2 (1.1)		158	19.3 (1.5)		59	7.4 (1.3)		20	2.8 (0.8)		51	6.5 (1.1)	
**Income**				0.0002			0.0018			0.1709			0.3603			0.5537			0.0949			0.1066			0.1612
Low	1200	443	39.4 (2.2)		302	69.6 (2.4)		204	65.5 (2.9)		28	11.0 (1.9)		70	23.5 (3.1)		35	11.8 (2.3)		16	4.1 (1.2)		38	11.4 (2.1)	
Low-average	1367	557	42.5 (1.8)		399	72.7 (2.4)		283	70.6 (2.7)		31	8.5 (1.7)		85	20.9 (2.4)		41	9.6 (1.7)		23	6.4 (1.4)		42	9.9 (1.9)	
High-average	1212	525	47.6 (1.7)		345	65.3 (2.0)		266	71.3 (3.9)		23	9.2 (2.0)		56	19.5 (3.5)		21	5.1 (1.5)		12	3.0 (1.2)		23	5.2 (1.4)	
High	1258	655	52.5 (1.4)		467	75.9 (2.3)		352	74.6 (2.1)		29	6.8 (1.4)		86	18.6 (1.8)		28	6.3 (1.2)		8	2.1 (0.9)		29	6.8 (1.8)	
**Employment status**				<0.0001			<0.0001			0.0031			0.0001			0.0553			0.002			0.072			0.0060
Working (including student)	3086	1539	51.0 (1.1)		1095	72.7 (1.4)		791	70.7 (1.6)		87	9.1 (1.0)		217	20.2 (1.5)		80	6.7 (0.8)		33	2.7 (0.5)		84	6.8 (0.9)	
Retired and homemaker	1330	364	28.4 (1.2)		214	57.0 (3.4)		180	84.8 (3.8)		8	2.4 (0.9)		26	12.8 (3.7)		8	3.3 (1.6)		6	2.0 (0.7)		8	3.2 (1.4)	
Unemployed	621	277	47.2 (2.6)		204	76.8 (2.8)		134	63.1 (4.1)		16	10.6 (2.9)		54	26.3 (3.0)		37	17.6 (3.7)		20	10.4 (2.9)		40	18.0 (3.6)	
**Neighborhood Social Deprivation level**				0.0079			0.6253			<0.0001			0.0571			<0.0001			0.0443			0.016			0.0450
No+Low	1369	675	49.6 (1.3)		478	70.5 (3.1)		389	80.9 (1.5)		28	5.7 (1.2)		61	13.4 (1.7)		30	5.7 (1.3)		11	1.9 (0.6)		31	5.8 (1.2)	
Medium-low+Medium	1878	775	45.0 (1.3)		547	72.7 (1.2)		379	64.4 (2.5)		42	10.1 (1.8)		126	25.5 (2.5)		42	6.6 (1.6)		27	4.8 (0.9)		49	8.2 (1.5)	
High+Very-high	1729	709	42.5 (1.7)		477	70.5 (2.8)		328	67.4 (2.5)		41	10.4 (1.5)		108	22.2 (1.8)		53	12.5 (2.3)		21	4.9 (1.1)		52	10.9 (1.7)	
**Total**	5037	2180	45.6 (0.72)		1513	71.2 (1.5)		1105	71.0 (1.2)		111	8.6 (0.9)		297	20.3 (1.3)		125	7.9 (1.0)		59	3.8 (0.5)		132	8.1 (0.9)	

Data from the São Paulo Megacity Mental Health Survey (SPMHS), Brazil, 2005–2007 (n = 5037).

1 Among past-year users.

2 Among regular users.

When we examined HDLF and HDHF outcomes among the past-year regular users (RU), about 9% qualified as recent ‘heavy drinkers of lower frequency’ and about 20% qualified as ‘heavy drinkers of higher frequency’ (versus the complement of 71% non-HDLF/HDHF). Also conditioned on past-year RU, about 4%–8% qualified for these recently active DSM conditions: DSM-IV abuse (7.9%); DSM-IV dependence (3.8%); DSM-5 AUD (8.1%). In basic cross-tabulations, males were more likely than females to be past-year users, RU, and HDHF drinkers (all p<0.05), but were not more likely to qualify for DSM conditions (among RU).

Regarding the main focus on area-level NSD, with two exceptions (RU and HDLF), all alcohol outcomes were associated with NSD (p<0.05). NSD inverse associations were seen for past-year use and for non-heavy drinking among RU. Positive NSD associations were seen with higher frequency heavy drinking (HDHF) and with all three DSM conditions.

Among men who reported drinking in the past 12 months (62%), nearly 80% drank regularly. For women, 30% reported alcohol use in the previous year and among those around 60% consumed alcohol in a regular basis. There were also more men than women with heavy and frequent drinking patterns among regular users. Unexpectedly, such sex differences were no longer observed for non-heavy drinkers and heavy drinkers, DSM-IV abuse, DSM-IV dependence, and DSM-5 AUD.

For RU, there was a higher prevalence of heavy drinking patterns in younger cohorts than in the oldest cohort (55 years old and more). For example, about 25% of those aged 18–34 years drank in the HDHF pattern, whereas around 7% of those aged 55 or more engaged in those drinking patterns.

In Table 2, we compared men and women in terms of quantity and frequency of alcohol consumption, by drinking patterns. We note that men and women had the same modal frequency of consumption per week within each drinking pattern category (1–3 days a month for non-heavy drinkers, and 1–2 days a week for HDLF and HDHF). However, women surpassed men in the modal number of doses on a typical drinking day when drinking in heavy drinking patterns. For both HDLF men and women, and HDHF women, the median number of doses on a typical drinking day was 6. When drinking in the heavy and frequent pattern, men had the modal number of doses of 7.

**Table 2:** Comparison of non-heavy drinkers, heavy drinkers of lower frequency and heavy drinkers of higher frequency in terms of quantity and frequency of alcohol consumption, by sex, in the São Paulo Megacity Mental Health Survey (SPMHS).

Quantity and frequencyof alcohol consumption	Non-heavy drinkers(n = 1105)		Heavy drinkers of lowerfrequency, HDLF (n = 111)		Heavy drinkers of higherfrequency, HDHF (n = 297)	
	Men	Women	Men	Women	Men	Women
Modal frequency ofconsumption	1–3 daysper month	1–3 daysper month	1–2 daysper week	1–2 daysper week	1–2 daysper week	1–2 daysper week
Modal doses on a typicaldrinking day	2	1	5	6	5	6
Median number of doseson a typical drinking day	2	2	6	6	7	6

### Correlates of recently active alcohol use, drinking patterns, and AUD for total sample, males and females

#### Neighborhood social deprivation level

The bottom part of Table 3 shows covariate-adjusted NSD associations with past-year drinking and both forms of heavy drinking (p<0.05). Individuals in mid NSD neighborhoods are less likely to be past-year drinkers (p<0.05). Among RU, those in the mid to higher level NSD neighborhoods are more considerably likely to drink heavily (p<0.05). When we hold constant age and neighborhood social deprivation, the inverse association with low income that stands out such that regular drinkers of low education are under-represented among heavier drinkers at the low frequency level, and, under the same constraints, these drinkers with low education are neither over- nor under-represented among heavier frequent drinkers (p>0.05) (Table 3).

**Table 3:** Total sample: Estimated odds ratios (OR) linking alcohol outcomes with Neighborhood Social Deprivation and other suspected determinants.

Characteristics	Past-yearuse^a^	Regularuse^b^	Heavydrinking oflowerfrequency (HDLF)^c^	Heavy drinkingof higherfrequency(HDHF)^c^	Abuse^d^	Dependence^e^	DSM-5AUD^f^
	AOR(95% CI)	AOR(95% CI)	AOR(95% CI)	AOR(95% CI)	AOR(95% CI)	AOR(95% CI)	AOR(95% CI)
**Sex**							
Men	3.5 (2.9–4.2)‡	2.5 (1.9–3.4)‡	1.3 (0.6–2.5)	1.6 (1.1–2.5)*	1.7 (0.8–3.6)	1.6 (0.7–3.6)	1.5 (0.8–2.8)
Women	1.0	1.0	1.0	1.0	1.0	1.0	1.0
**Age, years**							
18–34	2.0 (1.6–2.6)‡	0.7 (0.5–1.1)	7.3 (2.7–19.9)†	5.9 (2.1–17.0)†	7.0 (2.4–20.0)†	2.0 (0.5–9.0)	2.3 (0.7–7.0)
35–54	1.7 (1.2–2.3)†	0.8 (0.5–1.2)	2.5 (0.8–7.1)	4.1 (1.4–12.7)*	6.8 (2.6–17.7)‡	1.9 (0.4–8.7)	2.8 (0.9–9.1)
55+	1.0	1.0	1.0	1.0	1.0	1.0	1.0
**Marital status**							
Previously married	1.4 (1.1–1.8)†	1.1 (0.7–1.8)	1.1 (0.5–2.5)	1.1 (0.8–1.5)	1.0 (0.5–2.2)	1.2 (0.5–3.1)	1.0 (0.5–2.0)
Never married	0.9 (0.7–1.1)	0.9 (0.6–1.2)	1.3 (0.7–2.4)	1.2 (0.8–1.8)	1.3 (0.7–2.5)	1.2 (0.4–3.3)	1.9 (1.0–3.4)*
Married/cohabiting	1.0	1.0	1.0	1.0	1.0	1.0	1.0
**Education**							
Low	0.8 (0.6–1.0)	0.9 (0.7–1.3)	0.4 (0.2–0.8)†	1.4 (0.7–2.6)	1.3 (0.7–2.5)	3.0 (1.2–7.5)*	2.2 (1.0–5.0)*
Low-average	1.0 (0.8–1.2)	1.0 (0.7–1.3)	0.5 (0.2–1.0)	1.1 (0.6–1.9)	1.1 (0.5–2.4)	1.1 (0.3–3.3)	1.6 (0.8–3.3)
High-average+High	1.0	1.0	1.0	1.0	1.0	1.0	1.0
**Income**							
Low	0.6 (0.4–0.9)†	0.8 (0.5–1.0)	1.8 (0.8–3.9)	0.9 (0.5–1.5)	1.0 (0.5–2.0)	0.9 (0.2–4.0)	0.9 (0.4–2.3)
Low-average	0.6 (0.5–0.8)†	0.8 (0.6–1.0)	1.1 (0.6–1.9)	0.8 (0.5–1.2)	0.9 (0.5–1.7)	1.8 (0.6–5.5)	0.9 (0.3–2.2)
High-average	0.8 (0.6–1.0)*	0.5 (0.4–0.7)‡	1.2 (0.6–2.4)	0.8 (0.5–1.4)	0.6 (0.3–1.2)	1.0 (0.2–4.0)	0.5 (0.2–1.2)
High	1.0	1.0	1.0	1.0	1.0	1.0	1.0
**Employment status**							
Working (including student)	1.3 (1.1–1.5)‡	1.6 (1.2–2.1)†	1.8 (0.7–4.5)	0.8 (0.4–1.7)	0.9 (0.3–3.0)	0.9 (0.3–2.6)	1.3 (0.4–3.8)
Unemployed	1.3 (1.0–1.8)*	2.2 (1.4–3.5)†	1.7 (0.4–6.5)	1.8 (0.7–4.5)	2.5 (1.0–6.8)	3.5 (0.7–16.2)	3.5 (1.1–11.3)*
Retired and homemaker	1.0	1.0	1.0	1.0	1.0	1.0	1.0
**Neighborhood Social Deprivation level**							
No+Low	1.0	1.0	1.0	1.0	1.0	1.0	1.0
Medium-low+Medium	0.8 (0.7–0.9)†	1.1 (0.8–1.4)	2.0 (1.1–3.8)*	2.1 (1.4–3.1)†	0.9 (0.4–2.1)	1.7 (0.8–3.9)	1.1 (0.5–2.2)
High+Very-high	0.7 (0.5–1.0)	1.0 (0.6–1.5)	2.1 (1.1–3.8)*	1.7 (1.2–2.3)†	1.6 (0.7–3.6)	1.5 (0.7–3.4)	1.3 (0.7–2.7)

Data from the São Paulo Megacity Mental Health Survey (SPMHS), Brazil, 2005–2007 (n = 5037).

*Reference categories: a) non-past year users; b) non-regular users; c) non-heavy drinkers; d) regular users who did not fulfill criteria for abuse; e) regular users who did not fulfill criteria for dependence; f) regular users who did not fulfill criteria for DSM-5 AUD.*

*AOR, adjusted odds-ratio; CI, confidence interval.*

*All OR were adjusted for sex and age.*

*p<0.05;

† p<0.01;

‡ p<0.001.

Table 4, bottom part, indicates the association for men with respect to NSD. It is possible to see statistically robust, but not exceptionally strong, covariate-adjusted NSD associations with DSM-IV alcohol dependence and with heavy drinking at high frequency. The pattern of estimates is such that the excess odds of these outcomes are found when the RU lives in a mid-range NSD neighborhood (p<0.05).

**Table 4:** Males: Estimated odds ratios (OR) linking alcohol outcomes with Neighborhood Social Deprivation and other suspected determinants.

Characteristics	Past-year use^a^ (n = 1330)	Regular use^b^ (n = 1040)	Heavy drinking oflower frequency,HDLF^c^ (n = 77)	Heavy drinking ofhigher frequency,HDHF^c^ (n = 219)	Abuse^d^ (n = 102)	Dependence^e^ (n = 45)	DSM-5 AUD^f^ (n = 108)
	AOR (95% CI)	AOR (95% CI)	AOR (95% CI)	AOR (95% CI)	AOR (95% CI)	AOR (95% CI)	AOR (95% CI)
**Age, years**							
18–34	1.3 (0.9–2.0)	0.7 (0.4–1.2)	40.0 (3.6–449.9)†	4.9 (1.6–15.2)†	12.9 (3.7–45.0)‡	2.0 (0.4–9.3)	2.4 (0.8–6.7)
35–54	1.2 (0.8–1.9)	07 (0.4–1.3)	9.9 (1.0–99.6)	2.7 (0.8–8.7)	8.2 (2.9–23.4)‡	1.5 (0.3–8.0)	2.0 (0.6–6.7)
55+	1.0	1.0	1.0	1.0	1.0	1.0	1.0
**Marital status**							
Previously married	1.3 (0.8–2.1)	1.1 (0.5–2.1)	0.3 (0.1–1.3)	1.0 (0.7–1.6)	1.4 (0.7–2.7)	1.3 (0.5–3.2)	1.3 (0.7–2.5)
Never married	0.8 (0.6–1.2)	0.7 (0.5–1.0)	1.4 (0.6–3.7)	1.2 (0.7–2.0)	0.8 (0.4–1.8)	1.1 (0.3–4.4)	1.3 (0.6–3.0)
Married/cohabiting	1.0	1.0	1.0	1.0	1.0	1.0	1.0
**Education**							
Low	1.0 (0.7–1.3)	1.1 (0.7–1.7)	0.2 (0.1–0.6)†	1.2 (0.7–2.3)	1.3 (0.6–2.6)	2.6 (0.9–7.3)	2.3 (0.9–6.0)
Low-average	0.9 (0.8–1.1)	1.4 (0.9–2.1)	0.6 (0.2–1.4)	1.1 (0.7–1.9)	1.1 (0.5–2.2)	0.9 (0.3–2.8)	1.6 (0.9–3.0)
High-average+High	1.0	1.0	1.0	1.0	1.0	1.0	1.0
**Income**							
Low	0.5 (0.3–0.7)†	0.5 (0.2–1.0)	2.1 (1.0–4.5)*	0.8 (0.4–1.6)	1.2 (0.6–2.4)	0.9 (0.1–5.6)	1.0 (0.4–2.9)
Low-average	0.6 (0.5–0.9)†	0.7 (0.5–1.1)	1.0 (0.5–2.2)	0.6 (0.4–1.0)	0.9 (0.5–1.6)	1.5 (0.4–5.0)	0.8 (0.3–2.2)
High-average	0.9 (0.7–1.3)	0.4 (0.3–0.7)*	1.5 (0.6–3.4)	0.9 (0.5–1.6)	0.5 (00.2–1.1)	0.6 (0.1–2.7)	0.5 (0.2–1.3)
High	1.0	1.0	1.0	1.0	1.0	1.0	1.0
**Employment status**							
Working (including student)	2.0 (1.4–2.8)‡	1.7 (0.9–3.3)	0.3 (0.03–2.4)	1.3 (0.4–4.3)	0.3 (0.1–1.2)	0.8 (0.1–5.7)	1.6 (0.3–8.6)
Unemployed	2.4 (1.6–3.6)‡	2.8 (1.2–6.2)*	0.2 (0.02–1.3)	1.5 (0.5–4.7)	1.0 (0.2–4.6)	3.1 (0.2–40.7)	4.3 (0.6–30.4)
Retired and homemaker	1.0	1.0	1.0	1.0	1.0	1.0	1.0
**Neighborhood Social Deprivation level**							
No+Low	1.0	1.0	1.0	1.0	1.0	1.0	1.0
Medium-low+Medium	1.0 (0.7–1.4)	1.0 (0.7–1.6)	1.6 (0.8–3.3)	2.1 (1.3–3.3)†	0.8 (0.3–2.3)	2.4 (1.2–4.9)*	1.1 (0.5–2.5)
High+Very-high	0.8 (0.6–1.2)	1.2 (0.6–2.1)	1.6 (0.5–5.0)	1.5 (0.9–2.5)	1.3 (0.6–3.1)	1.5 (0.5–4.0)	1.1 (0.6–2.2)

Data from the São Paulo Megacity Mental Health Survey (SPMHS), Brazil, 2005–2007 (n = 2187).

*Reference categories: a) non-past year users; b) non-regular users; c) non-heavy drinkers; d) regular users who did not fulfill criteria for abuse; e) regular users who did not fulfill criteria for dependence; f) regular users who did not fulfill criteria for DSM-5 AUD.*

*AOR, adjusted odds-ratio; CI, confidence interval.*

*All OR were adjusted for age.*

*p<0.05;

† *p<0.01;*

‡ *p<0.001.*

Table 5, bottom part, shows no covariate-adjusted NSD-dependence association for women, but it is noteworthy that women living in areas with lower NSD level are more likely to be past-year drinkers (p<0.05). For ‘regular drinking’ outcome among women, no association was observed for NSD (p>0.05). Nevertheless, among RU, women in the higher level NSD neighborhoods are more substantially likely to qualify as cases of both forms of heavy drinking (p<0.05). In addition, when the RU lives in a mid NSD neighborhood, there also are excess odds of HFD (p<0.05). As for DSM-IV abuse, the covariate adjusted excess odds are seen for female RU living in the neighborhoods with higher levels of neighborhood social deprivation (p<0.05).

**Table 5:** Females: Estimated odds ratios (OR) linking alcohol outcomes with Neighborhood Social Deprivation and other suspected determinants.

Characteristics	Past-year use^a^ (n = 850)	Regular use^b^ (n = 473)	Heavy drinking oflower frequency,HDLF^c^ (n = 34)	Heavy drinking ofhigher frequency,HDHF^c^ (n = 78)	Abuse^d^ (n = 23)	Dependence^e^ (n = 14)	DSM-5 AUD^f^ (n = 24)
	AOR (95% CI)	AOR (95% CI)	AOR (95% CI)	AOR (95% CI)	AOR (95% CI)	AOR (95% CI)	AOR (95% CI)
**Age, years**							
18–34	3.0 (1.8–4.8)‡	0.8 (0.5–1.5)	2.3 (0.4–12.0)	15.6 (3.1–78.1)‡	0.3 (0.1–1.2)	07 (0.2–3.4)	0.3 (0.1–1.2)
35–54	2.1 (1.3–3.4)‡	0.9 (0.5–1.5)	1.7 (0.4–8.1)	21.7 (4.3–111.2)‡	1.0	1.0	1.0
55+	1.0	1.0	1.0	1.0			
**Marital status**							
Previously married	1.6 (1.2–2.2)†	1.2 (0.7–2.0)	1.8 (0.5–5.8)	1.0 (0.5–1.9)	0.7 (0.1–3.3)	1.0 (0.3–3.5)	0.9 (0.3–3.0)
Never married	1.0 (0.8–1.4)	1.1 (0.6–1.9)	1.1 (0.3–4.1)	1.3 (0.5–3.5)	5.0 (1.2–21.0)*	1.8 (0.5–7.0)	6.3 (1.6–25.2)†
Married/cohabiting	1.0	1.0	1.0	1.0	1.0	1.0	1.0
**Education**							
Low	0.7 (0.4–0.9)‡	0.8 (0.5–1.2)	1.1 (0.3–4.2)	2.2 (0.7–7.0)	1.3 (0.3–6.3)	6.4 (1.3–31.4)*	1.9 (0.4–8.5)
Low-average	1.1 (0.8–1.4)	0.7 (0.4–1.1)	0.4 (0.1–1.8)	0.9 (0.2–3.5)	1.7 (0.1–18.1)	2.2 (0.3–17.8)	2.2 (0.2–20.3)
High-average+High	1.0	1.0	1.0	1.0	1.0	1.0	1.0
**Income**							
Low	0.8 (0.5–1.2)	1.1 (0.6–1.9)	1.1 (0.3–5.1)	1.2 (0.3–4.2)	0.7 (0.1–3.4)	0.4 (0.1–2.4)	0.7 (0.1–3.2)
Low-average	0.6 (0.4–0.8)*	0.8 (0.5–1.2)	1.2 (0.3–4.9)	1.8 (0.6–4.9)	1.3 (0.3–5.1)	1.5 (0.2–13.4)	1.6 (0.4–6.6)
High-average	0.7 (0.4–1.0)	0.7 (0.4–1.0)	0.8 (0.2–3.0)	0.7 (0.2–2.8)	0.9 (0.2–3.4)	1.0	0.8 (0.2–3.4)
High	1.0	1.0	1.0	1.0	1.0	1.0	1.0
**Employment status**							
Working (including student)	1.1 (0.9–1.4)	1.5 (1.0–2.2)*	3.1 (1.3–7.5)*	0.8 (0.3–2.0)	1.6 (0.2–10.6)	1.8 (0.5–5.9)	1.2 (0.3–6.0)
Unemployed	1.1 (0.7–1.6)	1.9 (1.1–3.1)*	6.1 (1.5–23.8)*	1.7 (0.5–5.9)	4.8 (0.9–26.8)	6.9 (0.7–66.5)	4.4 (0.9–22.3)
Retired and homemaker	1.0	1.0	1.0	1.0	1.0	1.0	1.0
**Neighborhood Social Deprivation level**							
No+Low	1.0	1.0	1.0	1.0	1.0	1.0	1.0
Medium-low+Medium	0.6 (0.4–0.8)‡	1.1 (0.8–1.5)	4.9 (0.9–25.6)	2.3 (1.0–5.2)*	1.4 (0.3–5.7)	0.4 (0.1–3.2)	1.4 (0.4–5.4)
High+Very-high	0.6 (0.4–0.9)‡	0.8 (0.5–1.4)	5.8 (1.0–33.9)*	2.4 (1.1–5.3)*	3.7 (1.0–13.6)*	2.6 (0.6–10.3)	3.2 (0.9–11.6)

Data from the São Paulo Megacity Mental Health Survey (SPMHS), Brazil, 2005–2007 (n = 2850).

*Reference categories: a) non-past year users; b) non-regular users; c) non-heavy drinkers; d) regular users who did not fulfill criteria for abuse; e) regular users who did not fulfill criteria for dependence; f) regular users who did not fulfill criteria for DSM-5 AUD.*

*AOR, adjusted odds-ratio; CI, confidence interval.*

*All OR were adjusted for age.*

*p<0.05;

† *p<0.01;*

‡ *p<0.001.*

#### Income

Study of Tables 3, 4 and 5, column by column, discloses statistically independent inverse associations of past-year drinking with low to high-average income among total sample, with low-average income among both men and women, and with low income among men (but not women), where high income is the reference category (p<0.05).

The only other statistically robust associations with income are seen in Tables 3′s and 4′s column on regular use among total sample and male past-year users and heavy drinking among male regular users, and in Table 5′s column on female past-year drinking. Here, it is the high-average income male drinkers who are less likely than the high income drinkers to be recently active regular users (p<0.05) and low income male regular users being more likely to drink heavily in a low frequency (p<0.05). As compared to high income women, the covariate-adjusted odds ratio shows that low-average income women are less likely to be past-year drinkers (p<0.05).

#### Education

Educational attainment is another individual-level SES indicator that shows no general strength as a correlate of alcohol outcomes with few exceptions. In the covariate-adjusted model for past-year drinking among women, it is the least well educated women who are under-represented, as compared to their better educated peers (p<0.05). Among women RU, low education is associated with excess odds of being an active alcohol dependence case (p<0.05). This is also observed for the total sample, which shows 2–3-fold excess odds of alcohol dependence and DSM-5 AUD among regular users with low education attainment as compared to their peers with higher education levels.

Education is not associated with past-year drinking among men, but it is associated with HDLF status among male RU. In Table 4 the most educated male RU have excess odds of low frequency heavy drinking (HDLF) (p<0.05). This last finding is also valid for the total sample (Table 3).

#### Employment status

Our third individual-level SES indicator is employment status, which seems to have more to do with the lower levels of drinking status [past-year drinking (total sample and male), RU among past-year drinkers (total sample, male and female), and HDLF among RU (female)]. The first column of Table 4 shows a 2-fold excess odds of past-year drinking among unemployed (and among currently employed) males, as compared to the formerly employed (p<0.05). The 2nd and third columns of Table 5 also highlight excess odds of alcohol outcomes among unemployed, here in comparison with the formerly employed and unpaid homemakers in the household: (1) among female past-year users, RU is associated with being unemployed (and also with being employed); (2) among female RU, these same employment status values are associated with our alcohol outcome called ‘heavy drinking of lower frequency’ (p<0.05). The only statistically robust employment status association involves DSM-5 AUD among total RU, which can be seen in the last column of Table 3, where there is an estimated 4-fold excess odds of AUD among RU who are unemployed as compared to formerly employed (p<0.05).

#### Marital status

As for marital status, there are no noteworthy associations with alcohol outcomes among males (Table 4). Among women and total sample, in a comparison with those who are married or cohabiting, it is the previously married who are modestly more likely to be past-year drinkers (p<0.05; Table 5, first column). Among the female RU, the never married are more likely to be cases of the alcohol-related disturbances in the form of DSM-IV abuse and DSM-5 AUD.

## Discussion

With all the strengths of a transversal epidemiological survey of a representative sample of the adult general population living in the São Paulo Metropolitan Area, Brazil, this is the first Brazilian study to explore alcohol outcomes such as DSM-5 AUD in relation to suspected influences measured at both individual and neighborhood area levels and the associations of these outcomes with social position and possible socioeconomic inequalities. Overall, the main finding of note may be that regular alcohol users showing alcohol-related disturbances are generally more often found where area-level neighborhood characteristics reflect social disadvantage, even when important individual-level covariates have been held constant. More specifically, living in less deprived neighborhoods was associated with being a past-year alcohol drinker, while living in more deprived areas was associated with heavy drinking and some alcohol-related disturbances. As noted below, we would rather not offer a causal interpretation of this main finding. Nonetheless, it may be pertinent that those living in disadvantaged neighborhoods, with social exclusion and deprivation, might be most exposed to stress, less coping resources, high density of alcohol outlets. As such, the disadvantages experienced by residents of these neighborhoods extend beyond the indicators captured in our NSD assessment, which may influence heavy drinking [46] and the occurrence of alcohol related problems [45], [47], [61]–[63].

Other findings deserve to be highlighted as well, starting with a secondary focal point – namely, the individual-level SES indicators. Unemployed individuals often showed excess odds of alcohol-related disturbance (DSM-5 AUD), as compared to formerly employed or homemakers. In sex-specific analyses, female regular drinkers in the workforce were more likely to qualify for one of the forms of heavy drinking. Education associations also were noted, although not always with a consistently interpretable pattern.

As for our tertiary focal points, marital status was generally unrelated to alcohol outcomes among men, but among women, it was the previously married women who were more likely to be past-year drinkers; it was the never married women who were more likely to qualify as active cases of DSM-IV alcohol abuse and for DSM-5 AUD. It should not be surprising that adults age 18–34 were found to be over-represented among past-year drinkers. Among these young adults with at least 12 drinks in the past year, both forms of heavy drinking showed excess odds, relative to older RU, as did DSM-IV alcohol abuse (but not alcohol dependence nor DSM-5 AUD).

The results on prevalence estimates also may be of interest. These estimates reveal that although half of the sample is abstinent, nearly 30% of the past-year regular users drink in a heavy drinking pattern. It is particularly worrisome that, among heavy drinkers, two-thirds reports drinking in this pattern frequently - three or more times in a month period. Another important finding is the male-female convergence in the prevalence of heavy drinking pattern, reinforced by the observation that women are surpassing men in terms of estimated modal doses of alcohol consumption and women have reached men in terms of modal frequency of consumption in both heavy drinking patterns. The phenomenon of convergence in Brazil seems to be more due to an increase in female consumption than a reduction in male drinking [64]. For past-year use and regular use, the male/female ratio is similar to what has usually being found in the literature, with men surpassing a group of high educated women who work outside home [65]–[67]. When considering only those who were past-year regular users the sex-specific prevalence estimates for drinking patterns and AUD did not differ appreciably.

We note that heavy drinking was a common drinking pattern, similar to what has been found in other recent Brazilian surveys [10], [16], [68]–[70]. This pattern is specifically frequent in young adults aged between 18–34 years, which exposes them to a range of risk behaviors with adverse short- and long-term consequences, from social and physical problems - such as hangovers or medical illnesses, unprotected sexual activity, alcohol-related car crashes and other unintentional injuries - to increased risk for AUD [16], [20], [71], [72].

The 12-month prevalence of any DSM-IV AUD (alcohol abuse or dependence) among regular drinkers was 9.1%, which is slightly lower than reports from nationwide Brazilian studies probably due to methodological and sample differences [10], [11] and greater than a national survey conducted in Australia with similar methods as ours (6%) [73]. Conversely, when the DSM-5 AUD criteria were considered, we found a prevalence of 8.1%, which is lower than other two recent studies examining the impact of the new DSM-5 criteria on the prevalence of AUD: 12.3% in US (among alcohol lifetime users) [74] and 9.7% in Australia (among regular users) [73]. It is possible to hypothesize that the changes in the constructs required for DSM-5 AUD diagnoses - namely the inclusion of craving and exclusion of legal problem’s criterion - could have reflected in the prevalence of AUD differentially across countries and cultures, for SPMA and US the impact was lighter than the one observed for Australia. Moreover, the new AUD DSM-5 criteria may have led the AUD prevalence to a more consistent estimate in our sample, considering that 15% of the positive cases for both DSM-IV AUD had concurrent onsets of abuse and dependence as showed in a previous SPMHS report [9].

In Brazil, public health campaigns mainly target drinking driving and AUD. However, specific preventive strategies might be more targeted in relation to subgroups showing higher prevalence of “at risk” drinking, and those living in social disadvantageous situations. Other considerations note studied here are the current living circumstances and social support network of the individuals with harmful drinking practices. Of course, counter-balancing enthusiasm for these targeted interventions is evidence that often it is interventions working at both the individual and population levels (alcohol taxation, restrictions on alcohol availability) that prove to be the most effective policy options [3], [75].

### Study strengths and limitations

Our study has three important strengths. First of all, this project is based on data from a large, representative sample of adults residing in the metropolitan area of São Paulo. Secondly, it examines distinct alcohol outcomes using conditional prevalences and studies both the DSM-5 AUD and DSM-IV abuse and dependence, which were independently assessed by using the ungated approach described elsewhere. Thirdly, this study contributes to the recent theoretical discussion about effects on drinking patterns due to individual SES and neighborhood level deprivation.

In spite of these strengths, some limitations should be considered. Despite the representativeness of our sample, it is restricted to residents of a large urbanized area, which precludes generalization of our findings to the general population living in rural settings of Brazil. Moreover, there was insufficient information to take race or ethnicity into account as covariate in the models because in the CIDI version questionnaire used herein the question about ethnicity was only asked for respondents who self-identified as minorities (n = 156). Thus, this study cannot shed much light on sources of causal variation in drinking outcomes in Brazil as might involve linkages of race or ethnicity with neighborhood social deprivation and SES. Our survey measurements were not perfect and did not include constructs that belong in conceptual models of linkages between SES and alcohol outcomes (e.g., stress; social support buffers; access, availability, and cost of alcohol). As another aspect of measurement, lay interviewers with five days of training may not have the ability to make a refined assessment of alcohol related problems, although we note the generally favorable results on validity of the alcohol and other drug use disorders diagnoses, as assessed using clinical reappraisal study designs since the mid-1980s [51], [76], [77].

With a transversal epidemiological survey design, this project has a major limitation that tempers interpretation of its main finding on NSD and alcohol outcomes. As has been recognized for more than 80 years, individuals with alcohol problems may have selective migration toward neighborhoods at the higher NSD levels. Alternately, it is possible for these cases to be left behind as others without problems migrate upwardly and out to neighborhoods with lower NSD levels. Moreover, we cannot state whether these cases occurred as a consequence of disadvantageous social conditions (a social causation hypothesis) or whether the alcohol outcomes caused neglect and a worsening of social conditions (a social selection hypothesis), which are alternatives that have been most thoroughly discussed in psychiatric epidemiology and allied social sciences [74], [78], [79].

Future research of a prospective and longitudinal character can explore what might prove to be causal mechanisms that account for the otherwise heterogeneous patterns of association and male-female variations observed across alcohol outcomes in this study. For example, among RU, the AUD-unemployment association is possibly strong enough to rule out the possibility of a spurious association, but even so, the unemployment might cause AUD or it might be caused by AUD, among various alternative mechanisms. In future studies, it also should be possible to examine in detail the potential roles of social exclusion and deprivation as part of the underlying causal mechanisms.

### Conclusions

In this study, we found an association between neighborhood socioeconomic deprivation with heavy drinking patterns and AUD. This project brings important contributions to the study of alcohol use patterns in Brazil where it is the first study to investigate area-level neighborhood socioeconomic deprivation (independent of household income and other SES indicators) in an effort to build evidence for public health alcohol policy development in this country. As noted above, access to alcohol may be a neglected component of NSD in the Brazilian area-level scaling approach, and we may find that higher NSD are those in which alcohol outlets are largely concentrated (as has been found elsewhere; [80]–[82]). If this also is the case in Brazil, one important policy measure would be reduce the number of outlets that sell alcohol in these neighborhoods; another measure might be an increase in taxes on alcohol, with revenues directed toward future project to improve alcohol prevention and treatment in our country. The implementation of such policies has proved successful on other countries [3], [75], and we are hopeful that these epidemiological findings may help guide future directions for public health work along these lines, not only in Brazil but also more globally.

## Supporting Information

Table S1 **Neighborhood Social Deprivation (NSD) level distribution of the total sample, men and women.** Data from the São Paulo Megacity Mental Health Survey (SPMHS), Brazil, 2005–2007.
(DOCX)
