# Supplementary material for: Drinking Patterns and Alcohol Use Disorders in São Paulo, Brazil: The Role of Neighborhood Social Deprivation and Socioeconomic Status
Source: PLoS One. 2014 Oct 1;9(10):e108355. doi: 10.1371/journal.pone.0108355 (PMC4182710; doi:10.1371/journal.pone.0108355)
Supplement: Table S1 — Neighborhood Social Deprivation (NSD) level distribution of the total sample, men and women. Data from the São Paulo Megacity Mental Health Survey (SPMHS), Brazil, 2005–2007. (DOCX) [file pone.0108355.s001.docx]

**Table S1. Neighborhood Social Deprivation level distribution of the total sample, men and women. Data from the São Paulo Megacity Mental Health Survey (SPMHS), Brazil, 2005-2007.**

| **Neighborhood Social Deprivation level** | **Total sample**  **(n = 4976)** | | | **Men**  **(n = 2160)** | | | **Women**  **(n =2816)** | | |
| --- | --- | --- | --- | --- | --- | --- | --- | --- | --- |
|  | **n** | **Weighted**  **% (SE)** | **Unweighted**  **% (SE)** | **n** | **Weighted**  **% (SE)** | **Unweighted**  **% (SE)** | **n** | **Weighted**  **% (SE)** | **Unweighted**  **% (SE)** |
| No+Low | 1369 | 31.7 (0.6) | 27.5 (0.4) | 592 | 31.4 (1.3) | 27.4 (0.8) | 777 | 32.1 (0.7) | 27.6 (0.6) |
| Medium-low+Medium | 1878 | 36.7 (0.8) | 37.7 (0.8) | 829 | 37.3 (1.2) | 38.4 (1.0) | 1049 | 36.0 (1.0) | 37.2 (1.0) |
| High+Very-high | 1729 | 31.6 (0.7) | 34.8 (0.8) | 739 | 31.3 (1.3) | 34.2 (1.2) | 990 | 31.9 (1.0) | 35.2 (1.1) |
